# Anastomotic leak after esophagectomy for cancer in enhanced recovery protocol versus conventional care: clinical characteristics and outcomes

**DOI:** 10.3389/fonc.2026.1868622

**Published:** 2026-09-04

**Authors:** Yuhao Wen, Yixuan Huang, Kangning Wang, Domenico Pourmolkara, Jialong Li, Chenghao Wang, Xuefeng Leng, Qiang Fang

**Affiliations:** 1 North Sichuan Medical College, Nanchong, China; 2 Department of Thoracic Surgery, Sichuan Clinical Research Center for Cancer, Sichuan Cancer Hospital and Institute, Sichuan Cancer Center, Affiliated Cancer Hospital of the University of Electronic Science and Technology of China, Chengdu, China; 3 Department of Thoracic Surgery, Public Health Clinical Center of Chengdu, Chengdu, China; 4 Department of Thoracic Surgery, University of Perugia Medical School, Ospedale Santa Maria della Misericordia, Perugia, Italy

**Keywords:** anastomotic leak, enhanced recovery after surgery, esophageal cancer, esophagectomy, non-tube no fasting

## Abstract

**Background:**

Anastomotic leak (AL) is the most concerning complication after esophagectomy in the Enhanced Recovery After Surgery (ERAS) protocol. This retrospective cohort study aimed to compare the clinical characteristics and outcomes of postoperative AL in esophageal cancer patients under the ERAS protocol versus those receiving conventional care. Strategies to enhance the safety of the ERAS protocol were explored.

**Methods:**

This retrospective study included patients with carcinoma of the esophagus or esophagogastric junction who underwent transthoracic esophagectomy with gastric conduit reconstruction and developed postoperative AL between January 2018 and August 2023.The “Non-Tube No Fasting” ERAS protocol was implemented in the ERAS group. Inverse probability of treatment weighting (IPTW) was used to balance baseline characteristics.

**Results:**

Of the 483 patients in the ERAS group and 1,385 patients in the conventional group who underwent transthoracic esophagectomy with gastric conduit reconstruction, 33 and 140 patients, respectively, were included in the final analysis. After IPTW adjustment, among the nine early clinical manifestations of acute leukemia (AL), only tachyarrhythmia showed a significantly higher cumulative incidence in the ERAS group (P < 0.001). Both before and after IPTW adjustment, no significant differences were observed in time to diagnosis, endoscopic defect size, classification (classified as early or delayed), or severity between the two groups. Similarly, no significant differences were found in postoperative length of stay, hospital readmission rate, intensive care unit (ICU) readmission rate and corresponding ICU length of stay, defect healing rate, defect healing time, or leak-related mortality (all P > 0.05).

**Conclusions:**

The “Non-Tube No Fasting” ERAS protocol is not associated with worse clinical characteristics or outcomes of AL compared with conventional care. Further attention is warranted for patient selection strategies and improved identification of delayed AL. These findings are preliminary and should be confirmed in future studies.

## Introduction

The Enhanced Recovery After Surgery (ERAS) protocol promotes perioperative functional recovery, reduces symptom burden, and improves quality of life based on Patient-Reported Outcome measures (1, 2). However, esophagectomy is associated with a high postoperative complication rate, ranging from 25% to 60% (3). Anastomotic leak (AL), one of the most serious complications, significantly affects postoperative length of stay and mortality (4, 5). Consequently, the risk and severity of AL are the most critical concerns within the framework of ERAS. Although the ERAS protocol is generally considered safe (6, 7), its specific impact on clinical characteristics and outcomes of AL has not been thoroughly investigated.

The core philosophy of the ERAS approach is to reduce perioperative physiological stress, lower the incidence of postoperative complications, and shorten the length of hospital stay. Different esophageal cancer centers have implemented distinct ERAS protocols (8, 9), and the most variable component among these protocols is whether to modify traditional practices, specifically: nasogastric decompression, enteral tube feeding and postoperative fasting. The “Non-Tube No Fasting” ERAS protocol breaks away from the traditional practices. During the operation, the insertion of nasogastric tubes and nasojejunal feeding tubes is omitted. Postoperatively, patients are permitted to start early oral feeding, which allows them to restore normal physiological functions more quickly. Its primary measures deviate significantly from conventional care. However, surgeons remain concerned that these measures could potentially compromise anastomotic healing and significantly worsen the clinical characteristics and outcomes of AL. Therefore, most versions of the ERAS protocol do not include all these components simultaneously. Recent studies on early oral feeding have consistently reported its benefits without providing detailed clinical outcomes (10, 11), whereas a study indicated an increase in the length of hospital stay and incidence of AL (12), still resulting in a lack of robust evidence for clinical adoption. Because of the unpredictable nature of AL, this version is applied cautiously in clinical practice.

The clinical outcomes of AL are the primary factors influencing surgeons’ acceptance of ERAS protocol. It is challenging to promote a new perioperative management protocol when clinical evidence primarily focuses on the AL rates. Although similar AL rates have been reported between ERAS and conventional perioperative care (13), the clinical characteristics and outcomes of AL under the “Non-Tube No Fasting” ERAS protocol have not yet been investigated. Our study was the first to compare the clinical characteristics and outcomes of AL after esophagectomy between esophageal cancer patients under the “Non-Tube No Fasting” ERAS protocol and those under conventional care. The aim was to provide additional evidence for this ERAS version and to explore specific strategies for enhancing its safety.

## Methods

### Patients

This single-center retrospective cohort study consecutively enrolled patients who underwent transthoracic esophagectomy with gastric conduit reconstruction for cancer and developed postoperative AL between January 2018 and August 2023. The inclusion criteria were: (1) histologically confirmed malignancy of the thoracic esophagus or gastroesophageal junction, (2) transthoracic esophagectomy (McKeown or Ivor-Lewis procedure) with cervical or intrathoracic anastomosis, (3) gastric conduit reconstruction, and (4) postoperative esophagogastric AL. The exclusion criteria were: (1) esophagectomy performed via an abdominal-only approach; (2) reconstruction using colon or jejunum; (3) staple-line leaks originating from the gastric conduit without involvement of the esophagogastric anastomosis; (4) AL secondary to salvage radiotherapy or tumor recurrence; and (5) suspected AL without definitive confirmatory evidence. Clinical information and parameters related to AL were collected from the REDCap (Research Electronic Data Capture) of the esophageal cancer-specific database or the electronic medical records at our center. The oncologic treatment strategy, including the indication for upfront surgery or neoadjuvant therapy, the specific regimen when neoadjuvant therapy is chosen, and the timing of surgery, was determined through discussion by the same multidisciplinary team, while the attending surgeon was responsible for establishing the perioperative management protocol for each patient. A “Non-Tube No Fasting” version was implemented for the ERAS group. This protocol encompassed several key elements: no perianastomotic drainage, no nasogastric decompression, no enteral tube feeding, restrictive perioperative fluid management, and a combination of early oral feeding and parenteral nutrition. The ERAS protocol and conventional care were described in detail in our previous studies (1, 14).

Cancer staging was performed in accordance with the eighth edition of the American Joint Committee on Cancer (AJCC) guidelines. This study was conducted following the ethical standards of the Helsinki Declaration. The Ethics Committee for Medical Research and New Medical Technology at Sichuan Cancer Hospital approved this study (approval number: SCCHEC-02-2024-197). This study was reported in accordance with the STROBE statement (15).

### Definitions of AL

AL was defined as a full-thickness defect at the esophagogastric anastomotic ring, confirmed by radiological imaging or endoscopy. Radiological criteria included contrast extravasation at the anastomotic site on esophagogram, or an air-fluid level or hydropneumothorax communicating with a discontinuous anastomotic line on computed tomography. Endoscopic confirmation requires direct visualization of the defect. In addition, the presence of food, saliva, or gastric contents in the thoracic drain or cervical wound was considered direct clinical evidence of a defect. However, this finding requires imaging or endoscopic localization to confirm that the defect originated from the anastomotic ring rather than the gastric conduit staple line. Three severity types were defined according to the Esophagectomy Complications Consensus Group (ECCG) (16). AL was classified into the early group and the delayed group based on the timing and clinical setting of diagnosis: early AL was defined as diagnosis occurring ≤ 7 days postoperatively during continuous hospitalization, while delayed AL was defined as diagnosis occurring > 7 days postoperatively during continuous hospitalization or after discharge.

### Management of AL

When early clinical manifestations raised a high suspicion of AL, an emergency chest computed tomography scan was promptly performed. Particular attention was given to the following specific signs: (1) Pneumatosis or air-fluid level near the anastomosis or in the thorax. (2) Disruption in the continuity of the anastomosis line. Endoscopy was used to confirm uncertain computed tomography diagnoses. The ERAS patients underwent endoscopic insertion of nasogastric decompression and nasojejunal feeding tubes. Concurrently, fasting, nutritional support, and anti-infection therapy were administered. A chest drain was inserted into the pneumothorax or hydrothorax as needed. Unstable respiratory or circulatory conditions indicated a need for readmission to the intensive care unit (ICU). The healing of AL was reassessed using endoscopy or an upper gastrointestinal series.

### Statistical analysis

The Chi-square test or Fisher’s exact test was used to compare differences in categorical variables. The independent samples Student’s t-test and Mann-Whitney U test were employed to assess continuous variables with normal and non-normal distributions, respectively. To minimize between-group differences, inverse probability of treatment weighting (IPTW) was applied to achieve balance in baseline covariates. Stabilized weights were employed, and balance was assessed using standardized mean differences (SMD), with SMD <0.1 considered well-balanced. We compared the incidence of early clinical manifestations before the diagnosis of AL between the two groups. Cumulative incidence functions (CIF) were used to estimate the cumulative incidence of each manifestation, CIF curves are presented for visualization, and between-group differences in the CIF curves were assessed using bootstrap-based integrated tests over the whole observation period. There were no missing data for the variables used in the main analyses. Statistical analyses were performed using R (version 4.4.2, R Foundation for Statistical Computing, Vienna, Austria) with the ‘survey’, ‘tableone’, and ‘adjustedCurves’ package. A two-tailed P-value of less than 0.05 was considered statistically significant.

## Results

### Patient characteristics

**Figure 1:**
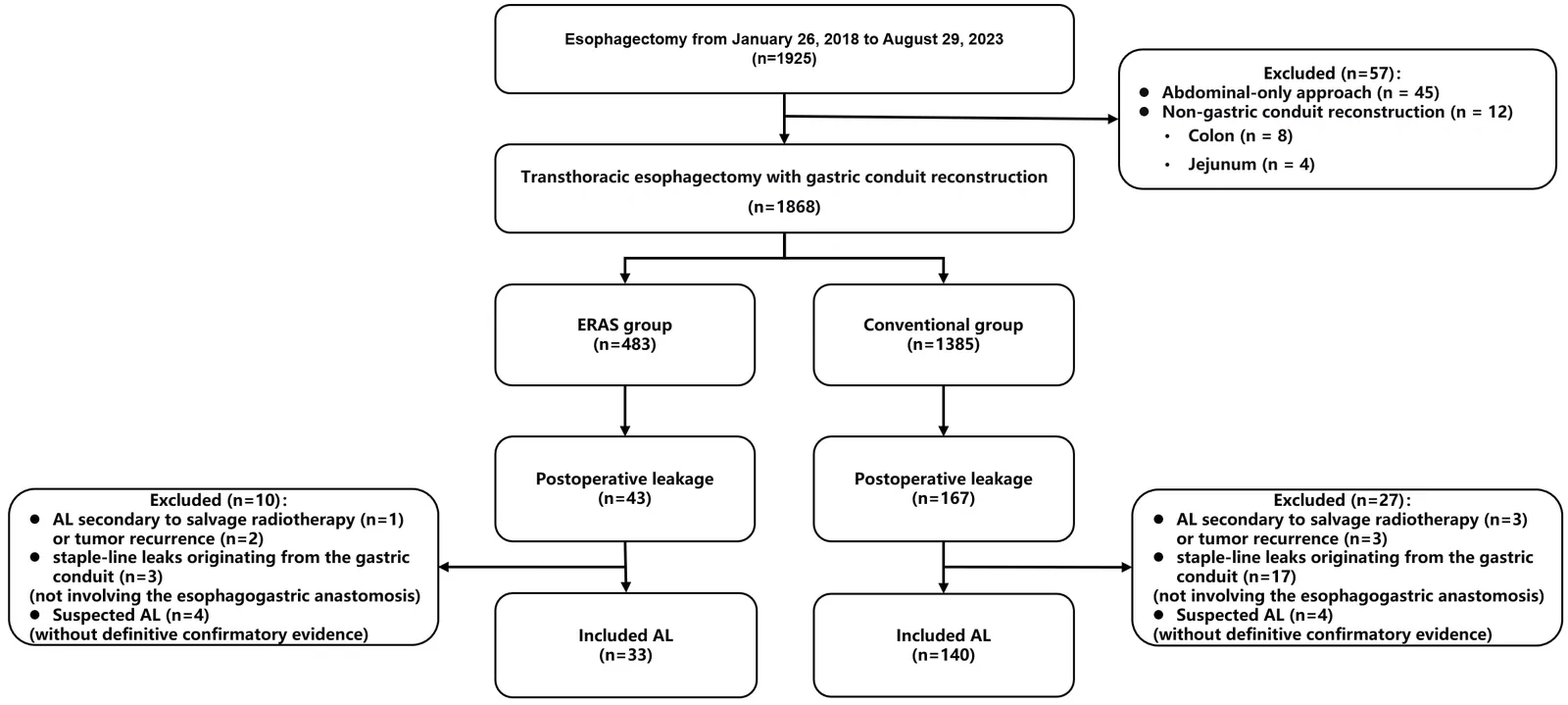
Flowchart of patient inclusion and exclusion. ERAS, Enhanced Recovery After Surgery; AL, anastomotic leak.

Among the 1,925 patients, a total of 1,868 underwent transthoracic esophagectomy with gastric conduit reconstruction: 483 patients were managed with the “Non-Tube No Fasting” ERAS protocol, and 1,385 received conventional care. Of these, AL occurred in 33 and 140 patients, respectively (**Figure 1**). Patient baseline characteristics before and after IPTW are summarized in **Table 1**. After IPTW adjustment, all baseline covariates were balanced between groups, with all post-weighting SMD values falling below the 0.1 threshold (**Supplementary Figure 1**).

**Table 1:** Baseline characteristics of enrolled patients before and after IPTW adjustment.

Variable	Unweighted				Weighted			
	ERAS group (n=33)	Conventional group (n=140)	p-value	SMD	ERAS group (n=31.5)	Conventional group (n=140.3)	p-value	SMD
Sex, n (%)			>0.999	0.004			0.745	0.069
Male	28 (84.8%)	119 (85.0%)			25.9 (82.3%)	119.1 (84.9%)		
Female	5 (15.2%)	21 (15.0%)			5.6 (17.7%)	21.2 (15.1%)		
Age, years, mean±SD	62.6±8.2	63.8±8.1	0.455	0.144	63.90±9.0	63.54±8.0	0.847	0.043
BMI, kg/m^2^, mean±SD	22.9±2.9	22.5±3.4	0.500	0.137	22.88±3.1	22.61±3.4	0.694	0.082
Smoking, n (%)			0.592	0.145			0.859	0.037
Yes	23 (69.7%)	88 (62.9%)			19.6 (62.2%)	89.8 (64.0%)		
No	10 (30.3%)	52 (37.1%)			11.9 (37.8%)	50.5 (36.0%)		
Drinking, n (%)			0.429	0.197			0.892	0.029
Yes	24 (72.7%)	89 (63.6%)			21.0 (66.7%)	91.8 (65.4%)		
No	9 (27.3%)	51 (36.4%)			10.5 (33.3%)	48.5 (34.6%)		
Comorbidities, n (%)			0.449	0.186			0.662	0.090
Yes	20 (60.6%)	72 (51.4%)			18.2 (57.9%)	74.9 (53.4%)		
No	13 (39.4%)	68 (48.6%)			13.3 (42.1%)	65.4 (46.6%)		
ASA score, n (%)			0.406	0.262			0.774	0.078
I-II	32 (97.0%)	127 (90.7%)			29.6 (93.9%)	128.9 (91.9%)		
III-V	1 (3.0%)	13 (9.3%)			1.9 (6.1%)	11.4 (8.1%)		
Neoadjuvant therapy, n (%)			>0.999	0.038			0.926	0.019
Yes	15 (45.5%)	61 (43.6%)			13.5 (43.0%)	61.7 (44.0%)		
No	18 (54.5%)	79 (56.4%)			18.0 (57.0%)	78.6 (56.0%)		
Surgical approach, n (%)			0.712	0.145			0.968	0.009
McKeown	31 (93.9%)	126 (90.0%)			28.5 (90.4%)	127.3 (90.7%)		
Ivor-Lewis	2 (6.1%)	14 (10.0%)			3.0 (9.6%)	13.0 (9.3%)		
MIE, n (%)			0.426	0.234			0.789	0.064
Yes	31 (93.9%)	122 (87.1%)			28.5 (90.4%)	124.2 (88.5%)		
No	2 (6.1%)	18 (12.9%)			3.0 (9.6%)	16.1 (11.5%)		
Anastomosis, n (%)			>0.999	0.067			0.713	0.088
Stapled	32 (97.0%)	135 (95.7%)			29.6 (93.9%)	134.5 (95.9%)		
Hand-sewn	1 (3.0%)	6 (4.3%)			1.9 (6.1%)	5.8 (4.1%)		
Histologic type, n (%)			>0.999	0.044			0.593	0.099
ESCC	31 (93.9%)	130 (92.9%)			30.1 (95.5%)	130.8 (93.2%)		
Non-ESCC	2 (6.1%)	10 (7.1%)			1.4 (4.5%)	9.5 (6.8%)		
Pathological stage, n (%)			>0.999	0.035			0.858	0.036
I-II	22 (66.7%)	91 (65.0%)			21.1 (67.0%)	91.6 (65.3%)		
III-V	11 (33.3%)	49 (35.0%)			10.4 (33.0%)	48.7 (34.7%)		

IPTW, inverse probability of treatment weighting; SMD, standardized mean difference; ERAS, Enhanced Recovery After Surgery; BMI, body mass index; ASA, American Society of Anesthesiologists; MIE, minimally invasive esophagectomy; ESCC, esophageal squamous cell carcinoma.

### Clinical characteristics

#### Early clinical manifestations of AL

**Figure 2:**
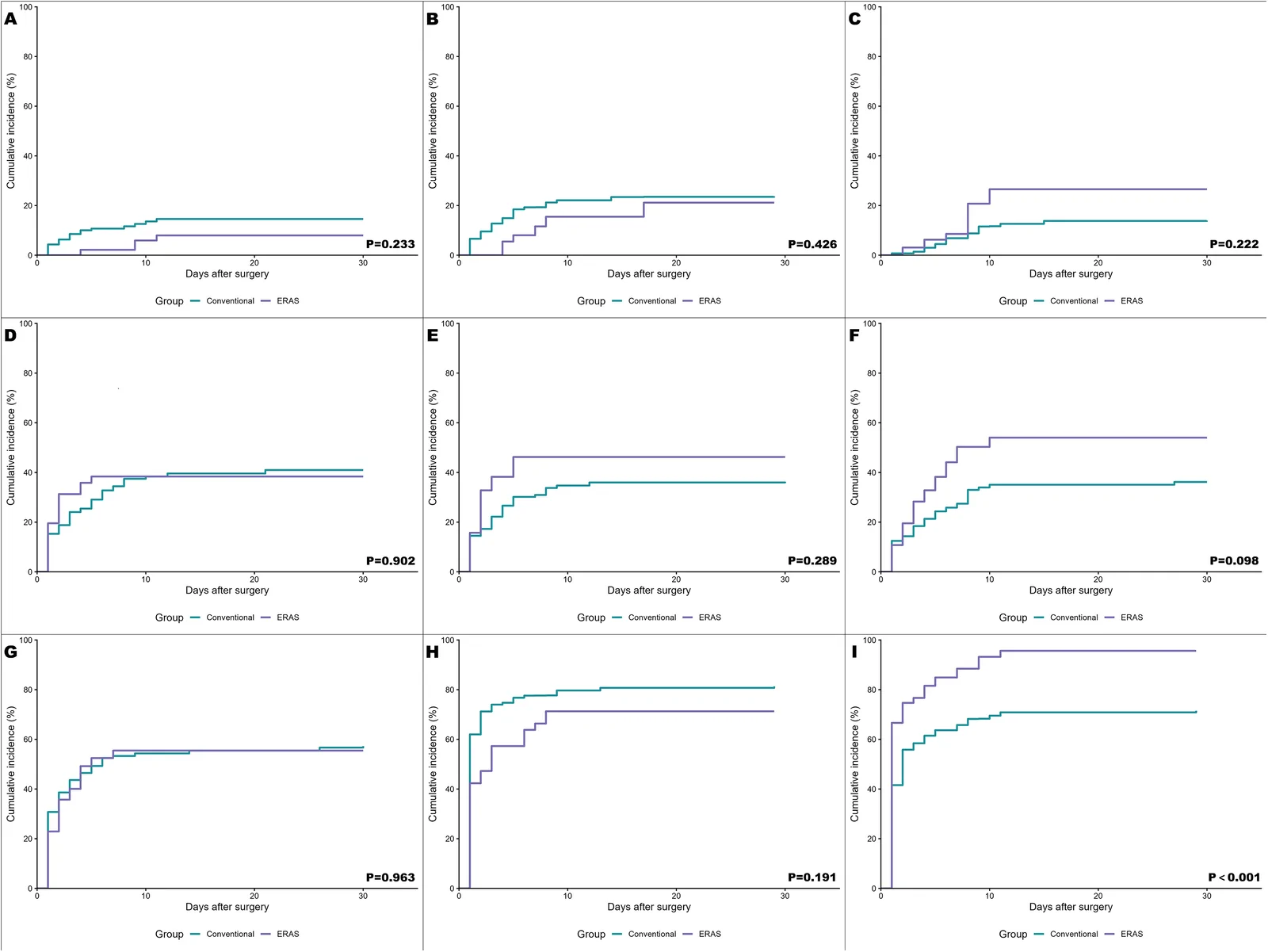
CIF curves of early clinical manifestations before the diagnosis of AL between the ERAS and conventional groups. Panels **(A)** through **(I)** present the CIF curves for nine prespecified early clinical manifestations: **(A)** purulent drainage, **(B)** dyspnea, **(C)** cervical wound infection, **(D)** cervical subcutaneous emphysema, **(E)** chest wall subcutaneous emphysema, **(F)** right pneumothorax, **(G)** fever (>38 °C), **(H)** leukocytosis, and **(I)** tachyarrhythmia. The x-axis represents days after surgery, and the y-axis represents the cumulative incidence (%). Green and purple lines denote the conventional group and the ERAS group, respectively. Between-group comparisons of the CIF curves were performed using bootstrap-based integrated tests over the whole observation period, following stabilized inverse probability of treatment weighting (IPTW). Among the nine manifestations, only tachyarrhythmia Panel **(I)** showed a statistically significant difference between the two groups (P < 0.001). The remaining eight panels **(A–H)** showed no significant between-group differences (all P > 0.05). CIF, cumulative incidence function. AL, anastomotic leak. ERAS, Enhanced Recovery After Surgery.

In the weighted analysis using stabilized IPTW, nine common early clinical manifestations were evaluated: fever, tachyarrhythmia, leukocytosis, cervical wound infection, cervical subcutaneous emphysema, dyspnea, chest wall subcutaneous emphysema, right pneumothorax, and purulent drainage. Among these, only tachyarrhythmia showed a statistically significant difference between the two groups in CIF curve comparison (P < 0.001), with a higher cumulative incidence in the ERAS group. The CIF curves of the remaining eight manifestations showed no significant difference between the two groups (all P > 0.05) (**Figure 2**).

### Characteristics of AL

**Figure 3:**
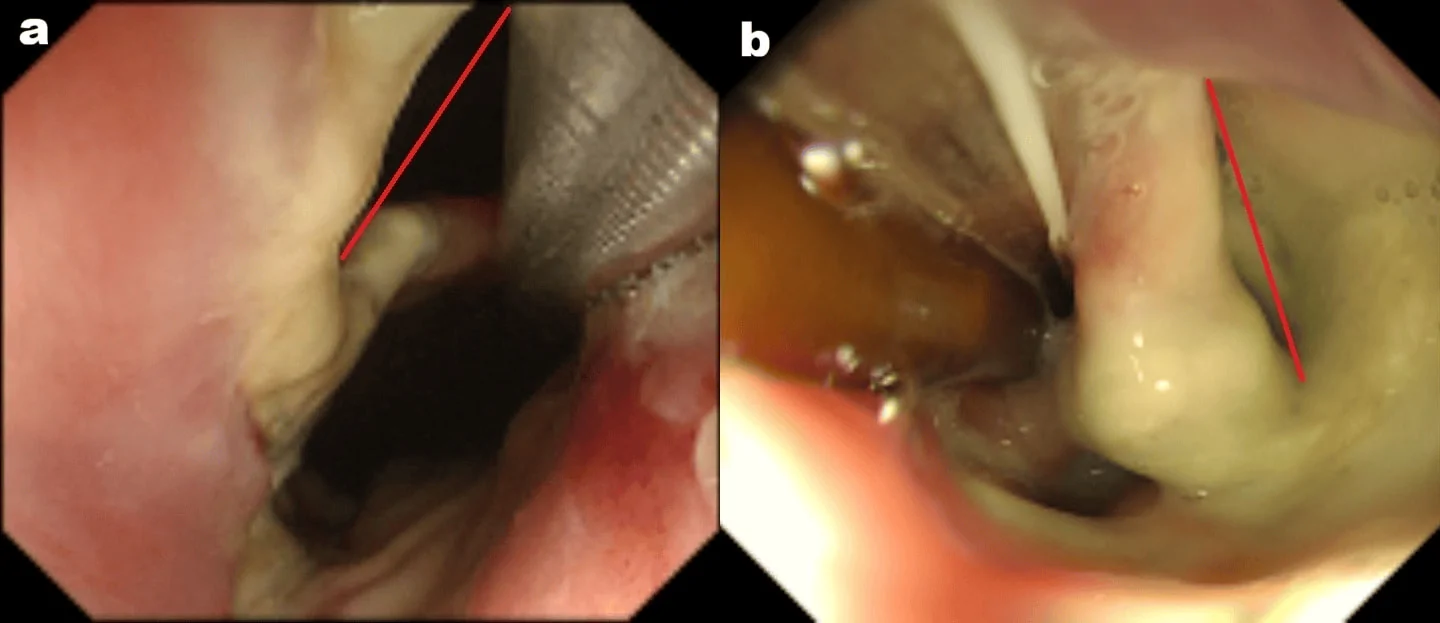
Representative endoscopic views of anastomotic defects in the ERAS and conventional groups. Panels **(a, b)** show representative endoscopic images of anastomotic defects in the ERAS group and the conventional group, respectively. The red lines indicate the long diameter of the defect, measured endoscopically as part of the routine assessment. Both panels demonstrate similar defect morphology and size between the two groups, which is consistent with the quantitative findings presented in **Table 2** (no significant difference in defect size between groups, P > 0.05). ERAS, Enhanced Recovery After Surgery.

The median time to diagnosis of AL was 9 days (IQR [interquartile range] 6-12) in the ERAS group and 10 days (IQR 7-14) in the conventional group, with no significant difference before or after IPTW adjustment (unweighted P = 0.298; weighted P = 0.468). Endoscopic defect size was measured in 168 out of 174 patients (**Figure 3**), and the results showed no significant difference between the two groups before or after weighting adjustment (both P>0.05) (**Table 2**). The proportions of early versus delayed AL and the severity of AL were similar between the two groups, and these findings remained consistent after weighting (all P>0.05) (**Table 2**). Additionally, the post-discharge AL rates were 27.3% in the ERAS group and 30.7% in the conventional group, with an unweighted P-value of 0.698 and a weighted P-value of 0.622.

**Table 2:** Comparison of AL characteristics between the ERAS and conventional groups before and after IPTW adjustment.

Variable	Unweighted			Weighted		
	ERAS group (n=33)	Conventional group (n=140)	p-value	ERAS group (n=31.5)	Conventional group (n=140.3)	p-value
Classification, n (%)			0.173			0.268
Early AL	15(45.5%)	46(32.9%)		13.7(43.5%)	46.1(32.8%)	
Delayed AL	18(54.5%)	94(67.1%)		17.8(56.5%)	94.2(67.2%)	
Defect size (long diameter), median (IQR), mm	7(4-10)	6(4-10)	0.826	6(4-10)	6(4-10)	0.762
ECCG type, n (%)			0.400			0.378
Type I	6(18.2%)	21(15.0%)		6.5(20.6%)	21.0(15.0%)	
Type II	27(81.8%)	112(80.0%)		25.0(79.4%)	112.1(79.9%)	
Type III	0(0.0%)	7(5.0%)		0(0.0%)	7.2(5.1%)	

AL, anastomotic leak; IPTW, inverse probability of treatment weighting; ERAS, Enhanced Recovery After Surgery; IQR, interquartile range; ECCG, Esophagectomy Complications Consensus Group. ECCG types: Type I, defect managed without change in therapy or with dietary modifications only; Type II, defect requiring interventional but not surgical treatment; Type III, defect requiring surgical intervention.

### Clinical outcomes

The two groups exhibited comparable clinical outcomes in terms of postoperative length of stay, hospital readmission rate, ICU readmission rate and ICU length of stay, defect healing rate and time to healing, and leak-related mortality, both before and after IPTW adjustment (all P > 0.05) (**Table 3**).

**Table 3:** Comparison of clinical outcomes between the ERAS and conventional groups before and after IPTW adjustment.

Variable	Unweighted			Weighted		
	ERAS group (n=33)	Conventional group (n=140)	p-value	ERAS group (n=31.5)	Conventional group (n=140.3)	p-value
Postoperative length of stay, median (IQR), d	21(9-36)	16(8-29)	0.133	22(9-38)	15(8-29)	0.059
Hospital readmission, n (%)	10(30.3%)	49(35.0%)	0.609	9.3(29.3%)	50.1(35.7%)	0.510
ICU readmission						
Rate, n (%)	7(21.2%)	27(19.3%)	0.802	7.9(25.1%)	27(19.2%)	0.491
Length of stay, median (IQR), d	2(1.5-6)	4(1.5-8.5)	0.389	2(2-7)	4(1-9)	0.301
Defect healing						
Rate, n (%)	30(90.9%)	130(92.9%)	0.703	29.2(92.5%)	130.8(93.2%)	0.874
Time, median (IQR), d	35(20-50)	42(26-60)	0.116	29(20-50)	41(25-58)	0.092
Leak-related mortality, n (%)	4(12.1%)	13(9.3%)	0.623	3.7(11.7%)	12.4(8.9%)	0.623

AL, anastomotic leak; IPTW, inverse probability of treatment weighting; ERAS, Enhanced Recovery After Surgery; IQR, interquartile range; ICU, intensive care unit.

## Discussion

The “Non-Tube” or “No Fasting” components within the ERAS protocol have become research focal points in recent years (10, 17, 18). However, these components have not been integrated into the general versions of ERAS following esophagectomy. A recent meta-analysis on the impact of early versus late oral feeding indicated that only four centers initiated early oral feeding on the first postoperative day (19). Despite the large volume of esophagectomies performed across healthcare centers in China, only two domestic centers have reported the routine implementation of the complete “Non-Tube No Fasting” ERAS protocol in existing literature (2, 14). The primary barrier is that the safety of AL under this comprehensive protocol has not been sufficiently assured. Previous reports have evaluated various components of the ERAS protocol individually, comparing different postoperative nutritional strategies, such as early versus late oral feeding (11, 19) or oral intake versus enteral tube feeding (20, 21), as well as the use versus omission of nasogastric tubes (18, 22). Several studies have also reported that the comprehensive “Non-Tube No Fasting” protocol does not increase the overall incidence of AL (1, 2). Notably, in the ERAS group, delayed AL accounted for more than half of all cases (54.5%), and 27.3% of cases were diagnosed after discharge. Although these proportions did not differ significantly from those in the conventional group, the high absolute rates associated with the ERAS protocol are clinically relevant. This significance stems from the fact that patients following this protocol rely primarily on oral feeding for nutritional support after discharge. Delayed AL in this setting may therefore carry increased risks if not promptly identified. Enhancing detection of delayed AL, particularly during the post-discharge period, is a critical step to improve the safety of the “Non-Tube No Fasting” ERAS protocol.

Our study showed that the ERAS group had a higher cumulative incidence of tachyarrhythmia before AL diagnosis. Tachyarrhythmia was observed in approximately 70% of patients in both groups. We speculate that this difference was primarily attributed to volume depletion in the ERAS group. Patients in this protocol initiated oral intake on the first postoperative day, and a perioperative restrictive fluid strategy was also implemented. The early inflammatory response triggered by AL further exacerbates volume depletion, and the discomfort associated with leakage often reduces appetite, thereby limiting oral fluid intake. In contrast, patients in the conventional group received more liberal fluid administration, which may have attenuated the hemodynamic response. Tachyarrhythmia was promptly alleviated with intravenous fluid resuscitation, supporting the conclusion that it originated from a hemodynamic cause. Apart from the higher cumulative incidence of tachyarrhythmia in the ERAS group, the overall profile of early clinical manifestations was similar between the two protocols. This finding indicates that the general strategy for early AL identification adopted in conventional care remains applicable in the ERAS protocol. When suspicious findings are identified, timely suspension of the ERAS protocol and transition to conventional management is warranted. Notably, all the ERAS AL patients developed at least one early clinical manifestation within the first three postoperative days, suggesting that the complete absence of any such signs during this period may help reassure clinicians that no complication has occurred. However, because patients following the ERAS protocol resume oral feeding on the first postoperative day, the occurrence of AL in this setting may lead to more severe consequences if not promptly identified. Therefore, even when early manifestations are non-specific, a lower threshold for diagnostic workup should be warranted in the ERAS group compared with the conventional care group. This approach aligns with the principle that early radiographic diagnosis is critical for the timely management of AL (23).In this study, the absence of a difference in the size of AL defects between the two groups suggests that the prompt discontinuation of the ERAS protocol, followed by the implementation of conventional management strategies, can effectively mitigate the progression of AL at early stage. Furthermore, no food residue was observed around the leakage site during the endoscopy in the early stage of AL. This is because the early warning signs were not triggered by food stimulation but by localized or systemic inflammatory responses due to poor anastomotic healing. The timely discontinuation of oral feeding, coupled with nasogastric decompression, can prevent leakage of gastrointestinal contents.

Previous studies on standard protocols have reported leak-related 30-day mortality rates ranging from 7.1% to 7.2% (4, 24), leak-related 90-day mortality rates from 11.3% to 11.7% (25, 26), and overall leak-related mortality rates from 5.7% to 34.8% (5, 27–30). In our cohort, the overall leak-related mortality rate in the ERAS group was 12.1% (4/33), which falls within the range of overall leak-related mortality rates reported in previous studies. Two of these deaths occurred within 30 days (6.1%), and the other two occurred between 30 and 90 days, resulting in a 90-day mortality rate of 12.1% (4/33). Because of the small number of events, IPTW-adjusted estimates for these endpoints could not be reliably calculated. Therefore, no statistical comparison between the two groups was performed for 30-day and 90-day mortality, and these rates are presented as unweighted descriptive data for the ERAS group only. Nevertheless, a detailed analysis of these deaths is essential to improve the safety of the ERAS protocol. One of the two 30-day deaths occurred in a patient with a history of bladder cancer surgery, diabetes, and long-term malnutrition (BMI 18.4 kg/m²), suggesting that patients with severely compromised baseline nutritional status may be unsuitable candidates for this ERAS protocol. The other 30-day death resulted from sudden-onset tension pneumothorax that developed during endoscopic treatment of anastomotic hemorrhage. Of the two additional deaths that occurred between 30 and 90 days, one involved a patient older than 75 years with significantly impaired pulmonary function. Although the AL had healed and the patient was discharged, death ultimately resulted from a recurrent pulmonary fungal infection. The other case involved a delayed AL, in which endoscopy revealed massive food retention in the mediastinum adjacent to blood vessels, which ultimately led to fatal hemorrhage. Because patients with delayed AL who continue oral feeding face higher mortality risks, it is a key safety measure to identify potential delayed AL before discharge.

The present study has some limitations. First, as a non-randomized controlled trial, perioperative management protocols and discharge criteria varied across surgical teams in line with their respective institutional preferences, which may have introduced selection bias. Nevertheless, the inclusion criteria were uniform for all patients, and surgical candidacy was determined by the same multidisciplinary team, which reduced potential bias in surgical decision-making. Second, variations in surgical techniques among different surgeons could have affected the characteristics and severity of AL. Third, although IPTW effectively balanced the measured baseline covariates, the possibility of residual confounding from unmeasured factors cannot be entirely excluded, as is the case for all observational studies. Notably, although the incidence of AL is relatively low, these cases were drawn from a large surgical cohort, which enhances the representativeness of our findings. Nevertheless, as with any non-randomized study, the large source population does not eliminate the inherent bias of this study design. Therefore, the generalizability of these findings to centers with different patient populations, surgical techniques, or perioperative management pathways requires further investigation.

## Conclusions

In conclusion, our study suggests that the “Non-Tube No Fasting” ERAS protocol is not associated with worse clinical characteristics or outcomes of AL compared with conventional care in patients undergoing esophagectomy for cancer. Our findings also indicate that careful patient selection and improved identification of delayed anastomotic leak may be key considerations for further enhancing the safety of this protocol in clinical practice. However, these results are preliminary and require confirmation in future studies.

## Data Availability

The raw data supporting the conclusions of this article will be made available by the authors, without undue reservation.

## Supplementary material

Supplementary Figure 1 Standardized mean differences (SMDs) of baseline covariates between the ERAS and conventional groups before and after inverse probability of treatment weighting (IPTW) adjustment. Each covariate is displayed on the y-axis, with corresponding SMD values plotted on the x-axis. Circles (•) = unweighted, triangles (▴) = weighted. The vertical dashed line at SMD = 0.1 indicates the conventional threshold for acceptable balance. Following IPTW adjustment, all covariates achieved SMD < 0.1, indicating successful balance between the two groups.
